# Prenatal determinants of fetal programming in dairy cattle: A mini-review

**DOI:** 10.3168/jdsc.2025-0926

**Published:** 2026-02-19

**Authors:** A.M. Oladele, M. Gobikrushanth, M. Oba, D.J. Ambrose

**Affiliations:** 1 Department of Agricultural, Food and Nutritional Science, University of Alberta, Edmonton, AB T6G 2P5, Canada; 2 Discipline of Veterinary Science, College of Science and Engineering, James Cook University, Townsville, QLD 4811, Australia; 3 Dairy Ecosystem Research and Development Center, Hiroshima University, Higashi-Hiroshima, 739-8528, Japan

## Abstract

Fetal programming in cattle is the result of the various maternal, paternal, and environmental determinants that influence prenatal development, affecting early postnatal life, adult life, and exerting possible transgenerational effects on the subsequent offspring. Prenatal environments establish long-term physiological trajectories through metabolic, endocrine, and epigenetic mechanisms, with consequences that may persist across generations. This review summarizes current knowledge about prenatal determinants that influence fetal programming in cattle and discusses economic implications. Maternal factors are the primary drivers of developmental programming. Maternal age, parity, level of milk production, nutrition, and health shape placental function, fetal growth, immunity, and reproductive capacity, and thereby creating trade-offs between offspring milk yield and fertility. Environmental heat stress (HS), particularly in late gestation, is among the most detrimental prenatal stressors, impairing growth, immunity, mammary development, fertility, and milk yield, with effects persisting across generations. Nutrient imbalance, metabolic stress, and gestational disease disrupt endocrine and epigenetic regulation, reducing growth efficiency, productivity, and longevity. Paternal factors, such as nutrition, age, and HS-induced epigenetic and transcriptomic changes in sperm, further modify embryonic development, often predisposing offspring to a metabolically conservative phenotype referred to as “economy mode.” Understanding these prenatal determinants is central to improving productivity, reproductive efficiency, and long-term herd survivability.

## Highlights

• Fetal programming is shaped mainly by maternal, paternal, and environmental factors.
• These factors cause epigenetic changes, permanently affecting calf development.
• In utero androgens and seminal plasma may influence fetal programming and calf performance.
• Suboptimal prenatal conditions affect not only immediate offspring but also future generations.

## Graphical Abstract

**Figure:**
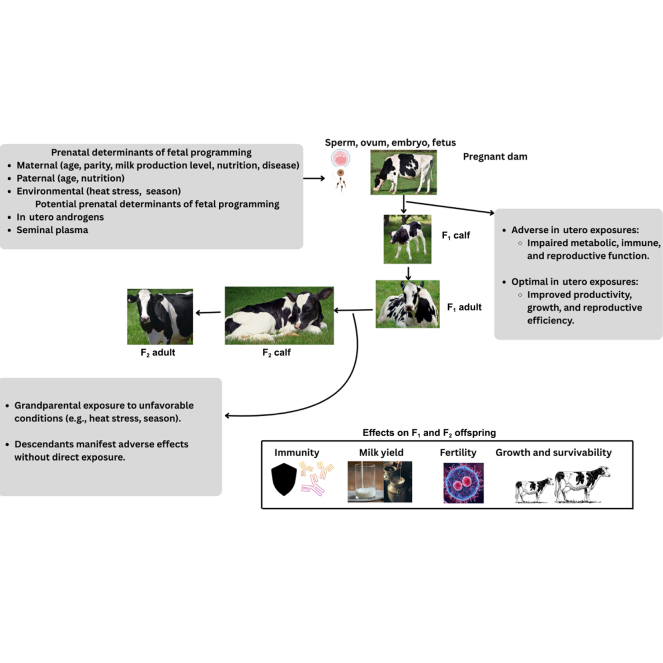
**Summary:** Fetal programming in cattle involves maternal, paternal, and environmental factors that shape prenatal development, influencing offspring traits across early life, adulthood, and potentially, future generations. This review examines the concept of fetal programming in cattle, where prenatal factors permanently influence offspring development. It presents evidence on key determinants, including maternal age, parity, milk production level, nutrition, and disease; paternal nutrition and age; and environmental stressors (e.g., heat stress). Prenatal androgen and seminal plasma are discussed as potential factors. These factors induce epigenetic modifications, shaping the calf's future health, productivity, and fertility. The evidence presented reveals that suboptimal prenatal conditions can lead to long-term consequences, including reduced milk yield and impaired reproduction, sometimes persisting across generations. Recognizing the negative impact of these prenatal determinants and taking mitigatory steps is essential for improving productivity, reproduction, and health, thereby supporting sustainable dairy farming.

Fetal programming is the result of the developmental imprints shaped by maternal, paternal, and environmental influences, affecting physiological, metabolic, and structural traits of the offspring (Johnston et al., 2008). It involves epigenetic modifications, immune system programming, and physiological changes (Nathanielsz et al., 2007). Epigenetics in livestock governs inheritable gene expression changes without altering DNA sequences, primarily via DNA methylation, histone modifications, and noncoding RNAs (Ibeagha-Awemu and Yu, 2021). Fetal growth integrates genetic and epigenetic factors, with prenatal conditions influencing adult health (Wu et al., 2006). The effects of fetal programming can be seen in the first filial generation (F_1_) and subsequent generations in animals. In human history, events such as the Dutch Hunger Winter and Chinese Famine have shown increased risks of psychiatric and metabolic disorders in exposed offspring (St Clair et al., 2005). Additionally, paternal smoking negatively affected sperm quality in sons (Kilama et al., 2024). In rats, maternal stress and advanced age program the offspring's health, affecting social behavior (Morley-Fletcher et al., 2004) and cardiovascular function (Shah et al., 2018). Maternal undernutrition in sheep has been consistently linked to altered fetal muscle and adipose tissue development, with consequences for growth and endocrine function later in life (Bonnet et al., 2010). Considering these effects in humans, rodents, and sheep, it is important to review the effects of prenatal determinants in domestic cattle.

Fetal programming in cattle increasingly affects fertility, productivity, and herd sustainability. Although prior reviews covered individual determinants such as maternal nutrition (Funston et al., 2012), heat stress (**HS**; Tao and Dahl, 2013; Cattaneo et al., 2022), or fertility outcomes (Wathes, 2022), no integrated analysis of maternal, paternal, and environmental influences exists. This mini-review consolidates evidence on how these factors shape offspring growth, productivity, fertility, immunity, survival, and transgenerational effects in dairy cattle. Key maternal determinants include age, parity, milk production level, nutrition, disease, and gestational HS, with maternal age strongly linked to reduced progeny milk yield.

Maternal age and parity have lasting programming effects on dairy offspring. Astiz et al. (2014) found that Holstein daughters with the highest lifetime milk yields (average of ∼14,300 L across 2 lactations) were born to younger dams (average 3 yr), compared with 4 yr for lower-yielding daughters, suggesting advantages associated with a younger maternal uterine and epigenetic environment. Younger dams are characterized by superior oocyte quality, lower cumulative metabolic and inflammatory burden, and a more favorable endocrine milieu, which may support more precise fetal programming of metabolic and mammary development (Astiz et al., 2014). Primiparous cows produce calves with reduced placental mass, lower birth weights, weaker vigor, higher mortality risk, impaired colostral IgG transfer, and reduced anti-Müllerian hormone (**AMH**; Bafandeh et al., 2023; Poczynek et al., 2023; Bobadilla et al., 2024). Daughters of older (12–16 yr) high-parity dams show enhanced longevity (Fuerst-Waltl et al., 2004), but very high-parity dams produce daughters that are less likely to reach exceptional lifetime yields (100,000 kg; Van Eetvelde et al., 2020). Bafandeh et al. (2023) reported that nulliparous and primiparous dams' daughters had higher milk yields (12,350 and 12,300 kg, respectively), possibly due to superior genetics for milk yield than young multiparous dams (2–6 parities) and old multiparous dams (7+ parities; 12,060 and 11,630 kg, respectively). However, young multiparous dams produced the heaviest calves (41 kg) with superior fertility and higher AMH, indicating greater ovarian reserves compared with daughters from nulliparous and primiparous dams. Thus, younger dams favor milk production, and older multiparous dams favor fertility.

Maternal milk yield during gestation programs offspring growth and reproductive performance through altered nutrient partitioning (Kamal et al., 2014). Extended lactation to 28 d precalving reduces daughters' DMI and early lactation milk yield, supporting a 45- to 60-d dry period to prevent fetal nutrient diversion (González-Recio et al., 2012; Kamal et al., 2014). Conversely, daughters of high-producing dams (≥14,000 to <16,000 kg/305 d) have higher birth weights, earlier heifer conception, elevated early-lactation fat-to-protein ratios, but longer postpartum calving-to-conception intervals due to metabolic strain despite normal AMH concentrations (Dehghan Harati et al., 2024). Thus, high maternal yield enhances fetal growth but compromises primiparous fertility.

Maternal nutrition during gestation shapes offspring growth, metabolism, and reproduction through lasting fetal programming. Although undernutrition impairs development, overnutrition is increasingly critical in high-producing dairy cows (Barcelos et al., 2022). Modern Holsteins face metabolic strain and negative energy balance near parturition, elevating nonesterified fatty acids and ketones that affect placental function and fetal epigenetics (Lemley, 2019; Martens, 2023). These stressors alter DNA methylation, permanently modifying genes involved in metabolism and immunity (Meesters et al., 2024). Timing matters in maternal nutrition as a determinant of fetal programming, as restricting nutrients to 65% of required energy intake during the first third of gestation reduced myofiber formation and IGF-1, limiting growth and intake (Noya et al., 2019; Meesters et al., 2024), whereas excess energy in late gestation triggered maternal lipolysis and ketonemia, programming fetal insulin resistance (Martens, 2023). Overnutrition also downregulates genes essential for testicular development and steroidogenesis, such as *StAR* (steroidogenic acute regulatory protein), *HSD17β3* (17β-hydroxysteroid dehydrogenase type 3), *IGF1*, *IGF2* (insulin-like growth factor 1, 2), and *IGF1R* (insulin-like growth factor 1 receptor), thereby disrupting fetal endocrine signaling (Weller et al., 2016). Targeted methyl donor supplements (choline, methionine) in late gestation improve placental and immune function via DNA methylation (Lemley, 2019). Both nutrient deficits and excess reduce offspring genetic potential, lowering milk yield, fertility, and longevity (Martens, 2023). Precision dry cow feeding that balances energy and micronutrients is key to optimal heifer performance (Drackley, 2011).

Maternal infection during gestation induces systemic inflammation that alters the intrauterine environment and programs long-term offspring deficits. Intramammary infection affects reproductive tissues and fetal development pathways (Campos et al., 2018), with epidemiological studies linking maternal mastitis to daughters' poorer health, fertility, and productivity (González-Recio et al., 2012; Swartz et al., 2021). Elevated maternal somatic cell count reduces daughter AMH concentrations, indicating diminished ovarian reserve (Ireland et al., 2011). Maternal illness also impairs colostrum quality and passive immunity transfer, increasing disease risk in early life, with lasting health and udder health consequences (Khan et al., 2024). Gestational infections thus persistently compromise immunity, fertility, and longevity.

Fetal exposure to maternal androgens, particularly testosterone and dihydrotestosterone, determines anogenital distance (**AGD**), a biomarker of androgen exposure during the reproductive programming window (Dean et al., 2012). In dairy cattle, AGD, measured from the anus to the base of the clitoris, is inversely associated with fertility, with shorter AGD linked to improved reproductive outcomes (Gobikrushanth et al., 2017; Carrelli et al., 2022), suggesting underlying endocrine and epigenetic programming during fetal gonadal differentiation. Seminal plasma is another potential determinant that can modulate developmental programming. Its bioactive components regulate endometrial receptivity and periconceptional embryo development, whereas sperm deliver epigenetic regulators, including small noncoding RNAs and retained histones, that influence early gene expression (Ostermeier et al., 2004; Krawetz, 2005). In Holstein heifers, intrauterine infusion of seminal plasma increased female calf birth weight when X-sorted semen was used, but not with conventional semen, likely due to prior exposure (Ortiz et al., 2019). With more evidence still needed, both maternal androgen exposure and seminal plasma are potential contributors to fetal programming in dairy cattle.

Although paternal prenatal factors are less studied than maternal factors, emerging evidence indicates that sire-related influences can shape fetal programming and postnatal outcomes in cattle. Bull age and nutrition are key paternal determinants influencing offspring outcomes, primarily through epigenetic modifications of sperm. Sire age has also emerged as a relevant programming factor. The use of very young genomic sires introduces sperm with distinct methylation patterns and telomere characteristics that may influence embryonic development and postnatal performance (Lambert et al., 2018), whereas aging sires transmit age-related epigenetic changes associated with poorer offspring health. Notably, daughters of older bulls (>1,500 d at semen collection) exhibit higher SCS than those of younger sires (<400 d), indicating compromised udder health and immune resilience (Fouéré et al., 2024). Together, these findings suggest that both very young and older sires can transmit age-dependent epigenetic signatures affecting offspring development. Among paternal factors, HS is the most pervasive in dairy systems. Paternal HS disrupts the blood–testis barrier and increases reactive oxygen species, leading to sperm DNA damage and altered transcriptomic profiles (Dahlen et al., 2023). These changes give rise to embryos exhibiting an “economy mode” phenotype, characterized by reduced cellular activity, mitochondrial dysfunction, and altered gene expression and epigenetic regulation (Sirard, 2025). Although this phenotype may enhance short-term survival under stress, it is associated with long-term reductions in milk yield, growth, immune function, and survival, with effects that can persist across generations, underscoring the significance of paternal programming in cattle.

Nutritional status modulates sperm-mediated programming; for instance, n-3 fatty acid supplementation improves sperm quality and blastocyst development and increases embryonic *OCT4* (octamer-binding transcription factor 4) and *PLAC8* (placenta-specific 8) expression (Goissis et al., 2013; Kalo et al., 2022), although links to long-term postnatal performance remain limited. Stronger intergenerational evidence comes from dietary manipulation of one-carbon metabolism: prepubertal rumen-protected methionine supplementation in sheep altered sperm DNA methylation and was associated with reduced body weight and scrotal circumference at puberty in male offspring, without affecting pubertal timing (Gross et al., 2020). Collectively, these findings indicate that perturbations in dietary methyl donors such as methionine or folate can induce heritable sperm epigenetic changes that predispose offspring to metabolic dysregulation, with implications for feed efficiency and body condition (Sinclair et al., 2016; Graybeal, 2025).

Maternal HS during gestation, programs long-term deficits in offspring growth, immunity, fertility, and milk production, with effects dependent on timing of gestational heat stress exposure. Late-gestation HS (final 45 d) consistently reduces birth and weaning weights, neonatal immunity, first-lactation survival, and milk yield (Tao et al., 2012; Monteiro et al., 2016). This arises from thermoregulatory blood flow redistribution compromising uterine perfusion and placental efficiency (Tao et al., 2012). The dry period is critical due to rapid fetal mammary development, where elevated maternal cortisol and catecholamines suppress fetal estrogen receptor α (**ERα**) expression, disrupting ductal morphogenesis, reducing secretory parenchyma, and increasing adipose deposition (Larsen and Laporta, 2024; Laporta and Guenther, 2025). Daughters exposed to HS in the final 2 mo of gestation show reduced mammary tissue independent of birth weight (Dado-Senn et al., 2022; Laporta and Guenther, 2025). In utero oxidative stress disrupts thymic and splenic development and postnatal colostral IgG absorption, increasing the risk of pneumonia and scours (Monteiro et al., 2016). Using data from 172,905 Holstein cows, Kipp et al. (2021) showed that elevated temperature-humidity index (**THI**) during the final 8 wk of gestation adversely affected daughter performance on a timing-dependent manner. Based on the THI ≥60 threshold linked to reduced milk yield and elevated SCS (Brügemann et al., 2012), HS during the final 1 to 3 wk prepartum reduced daughter milk yield, whereas exposure 5 to 8 wk prepartum produced a small compensatory increase. However, THI ≥50 across the entire 8-wk period consistently reduced milk fat percentage and impaired reproductive performance, as indicated by lower 56-d nonreturn rates and prolonged calving-to-first-insemination intervals (Kipp et al., 2021).

Transgenerational consequences of late-gestation HS are pronounced. In a decade-long retrospective study, Laporta et al. (2020) reported that Holstein dams exposed to HS during the final 46 d of gestation produced F_1_ daughters with shorter lifespans, reduced productive life, greater stillbirth rates, and lower milk yield across 3 lactations compared with daughters of cooled dams. Although reproductive traits were largely unaffected in F_1_, deficits intensified in the F_2_ generation, with granddaughters exhibiting reduced survival, increased stillbirths, and poorer lactational performance. These multigenerational losses impose an estimated $600 million annual economic burden in the United States, driven by reduced milk yield, increased rearing costs, and shortened herd longevity (Laporta et al., 2020).

Mechanistically, Larsen and Laporta (2024) linked these transgenerational effects to early mammary developmental disruption in F_2_ heifers born to heat-stressed F_1_ Holstein dams, characterized by reduced parenchymal area, fewer epithelial structures, decreased proliferation, increased apoptosis, and markedly lower ERα expression by 70 d of age, despite normal growth and intake. Complementary evidence from Macciotta et al. (2023) shows that seasonal thermal exposure of great-granddams influences milk yield and estimated breeding values in great-granddaughters, implicating stable epigenetic mechanisms, including DNA methylation. Collectively, these findings demonstrate that gestational and seasonal HS imprints performance across multiple generations, complicating genetic evaluation and underscoring the importance of mitigating thermal stress in dry cows.

Fetal programming in cattle is determined by a convergence of maternal physiological states (such as age, nutrition, and disease), paternal influences, and environmental stressors such as HS, which collectively dictate offspring health, reproduction, and productivity (Table 1). Although most evidence summarized here is derived from dairy cattle, mechanistic understanding of prenatal programming is informed largely by smaller ruminant and rodent model systems. Across maternal, paternal, and environmental influences, these studies show that prenatal perturbations induce persistent epigenetic modifications, with DNA methylation acting as a primary mechanism by stably regulating gene transcription in response to in utero conditions, alongside histone modifications and noncoding RNA regulation. Limited cattle data linking these exposures to altered offspring gene expression and developmental outcomes support the biological relevance of these conserved epigenetic mechanisms in dairy cattle. Consequently, future research must prioritize identifying these epigenetic markers and quantifying their transgenerational economic impacts to enable management strategies that maximize lifetime herd profitability.

**Table 1:** Prenatal determinants that affect fetal programming in cattle, their origin, proposed mechanism(s), and the direction of effect on the offspring

Origin	Determinants	Proposed mechanism	Direction of effect on daughter	Citation(s)
Maternal	Age and parity (younger dams)	Superior oocyte quality and favorable endocrine milieu, but reduced placental mass and nutrient transfer.	↑ Milk yield ↓Birth weight and immunity ↓Ovarian reserve (AMH)	Astiz et al. (2014); Bafandeh et al. (2023); Bobadilla et al. (2024)
	Age and parity (older and multiparous dams)	Higher cumulative metabolic and inflammatory burden, but greater placental capacity.	↑ Fertility and longevity ↓Peak lifetime yield potential	Fuerst-Waltl et al. (2004); Van Eetvelde et al. (2020); Bafandeh et al. (2023)
	Milk production (extended lactation), high milk production	Altered nutrient partitioning (competition between mammary gland and fetus) and postpartum metabolic strain.	↓ DMI and milk yield ↑Birth weight and reproductive maturity ↓ Fertility (due to high metabolic demand)	Kamal et al. (2014) Dehghan Haratiet al. (2024)
	Nutrition (overnutrition-high energy)	Lipolysis and ketonemia induce fetal epigenetic stress; downregulation of testicular steroidogenic genes.	↑Fetal insulin resistance ↓Testicular development (males)	Weller et al. (2016); Martens (2023)
	Nutrition (methyl donors)	Dietary methyl donors (methionine and choline) modify DNA methylation patterns.	↑Placental function ↑Immune and metabolic development	Lemley (2019); Meesters et al. (2024)
	Infection and inflammation (e.g., mastitis)	Systemic inflammation disrupts metabolism; extramammary effects alter gene expression in reproductive tissues, affecting placental function pathways.	↓Daughter milk yield ↓Ovarian reserve (AMH) and fertility ↓Passive immunity	Ireland et al. (2011); González-Recio et al. (2012); Campos et al. (2018); Swartz et al. (2021);
	Hormonal environment (androgens)	Exposure to maternal androgens programs gonadal differentiation; anogenital distance (AGD) serves as a biomarker.	Short AGD daughters: ↑Fertility Long AGD daughters: ↓Fertility	Gobikrushanth et al. (2017); Carrelli et al. (2022)
	Heat stress (mammary development)	Blood flow diversion to skin causes fetal hypoxia; oxidative stress impairs lymphoid organ development. Fetal cortisol/catecholamines suppress fetal mammary ERα expression; impaired ductal morphogenesis.	↓Birth weight ↓IgG absorption (passive transfer) ↑Disease susceptibility ↓Milk yield (F_1_ and F_2_ generations) ↓Productive lifespan	Tao et al. (2012); Monteiro et al. (2016); Laporta et al. (2020); Larsen and Laporta (2024)
Paternal	Nutrition (n-3 fatty acids and methyl donors)	Altered sperm epigenetics (DNA methylation) and blastocyst gene expression (e.g., *OCT4*, *PLAC8*).	n-3 Fatty acids: ↑Embryo competence Methyl deficiency: ↓Offspring insulin resistance	Goissis et al. (2013); Sinclair et al. (2016)
	Age (old sires)	Accumulation of sperm DNA methylation errors and age-related epigenetic changes.	↓Daughter udder health ↑Somatic cell score	Fouéré et al. (2024); Lambert et al. (2018)
	Heat stress	Reactive oxygen species induce sperm DNA damage; embryos adopt “economy mode” (mitochondrial dysfunction and altered transcriptomics).	↓Immune resilience ↓Milk yield and growth ↓Immune function and survival	Dahlen et al. (2023); Sirard (2025)
